# Optical coherence tomography angiography in retinitis pigmentosa: A narrative review

**DOI:** 10.1097/MD.0000000000030068

**Published:** 2022-08-26

**Authors:** Bingwen Lu, Guojun Chao, Like Xie

**Affiliations:** Department of Ophthalmology, Eye Hospital, China Academy of Chinese Medical Sciences, Beijing, China

**Keywords:** macular microvasculature, optical coherence tomography angiography, retinal biomarkers, retinal function, retinitis pigmentosa, vessel density

## Abstract

Retinitis pigmentosa (RP) is a group of inherited retinal disorders characterized by progressive rod and cone photoreceptor degeneration. Changes in retinal vasculature have long been associated with RP. Optical coherence tomography angiography (OCTA) is a novel imaging technology that enables noninvasive visualization of the retinal and choroidal microvasculature. OCTA enables quantification of microvascular changes in the retinal capillary plexus and choriocapillaris, in addition to qualitative feature description. Therefore, OCTA has the potential to become an important tool for better understanding, early detection, progression, and treatment of RP. In this review, we focus on the applications of OCTA in clinical research on RP. We also discuss future improvements in the OCTA technology for RP management. We believe that the advancement of the OCTA technique will ultimately lead to a better understanding of RP and aid in the prevention of visual impairment.

## 1. Introduction

Retinitis pigmentosa (RP) is a group of genetically diverse inherited retinal dystrophies characterized by progressive degeneration of rod and cone photoreceptors.^[1]^ RP patients usually suffer from impaired dark adaption, night blindness, visual field constriction, and central vision deterioration around 40 years of age.^[2,3]^ The relationship between ocular hemodynamics and RP has not been fully understood. Previous studies have confirmed the reductions in blood flow in RP patients,^[4]^ questions have yet to be answered regarding the role of vascular dysfunction, as well as vascular abnormalities in the foveal and parafoveal regions in the degeneration of photoreceptors. Owing to recent advances in imaging technology, optical coherence tomography angiography (OCTA) enables non-invasive visualization and quantitative assessment of the retinal and choroidal microvasculature, which shows great potential in providing diagnostic, prognostic, and perhaps therapeutic biomarkers of ocular hemodynamics in RP patients.^[5]^

Our purpose is to conduct a database search of all published studies that focus on the use of OCTA in clinical research on RP to better understand this retinal disorder. Further, we envision that advancements in OCTA technology could help clinicians in RP diagnosis, follow-up, and treatment in the near future.

## 2. Methods

A literature review was performed using PubMed, including all original studies registered until February 2021. Using the keyword “RP” and “OCTA”, 38 relevant publications published from 2016 to 2021 were retrieved.

Ethics committee approval was not requested because it is not needed for narrative reviews of the literature.

## 3. Current methods for retinal vasculature evaluation in RP

Evaluation of retinal vasculature was historically dependent on fluorescein angiography (FA), which could demonstrate abnormal retinal and choroidal vasculature in patients with RP, including prolonged transit time, narrowed vessels, and lower dye concentration.^[6]^ However, FA is invasive and has been reported to cause hepatic, renal, or allergic complications due to the need for intravenous injection and the possibility of leakage of dye.^[7]^ Fundus autofluorescence (FAF) is also useful in evaluating the retinal status in RP patients. Photoreceptor loss corresponds to hypo-autofluorescence in the peripheral fundus, whereas areas of hyper-autofluorescence correspond to increased lipofuscin in the retinal pigment epithelium (RPE).^[8]^ However, FAF produces a lower signal strength than FA and is monochromatic which does not produce multicolored images.^[9]^ Optical coherence tomography (OCT) has been used to evaluate RP to reveal decreased foveal thickness, interruption of the photoreceptor inner/outer segment (IS/OS) junction, and significantly reduced choroidal thickness.^[4,10]^ OCT structural changes correlate with the deterioration of sight.^[11]^ While these measurements are useful, OCT does not possess the ability to visualize microvascular changes during RP progression. The above modalities provide information about the structures of interest but do not provide details of vascular structure and blood flow.

OCTA is a recent technological advancement that allows for the acquisition of high-resolution, depth-resolved retinal images of both retinal and choroidal vascular layers in a rapid, non-invasive manner.^[12]^ With the advantages of differentiating the superficial capillary plexus (SCP), deep capillary plexus (DCP), and choriocapillaris plexus (CCP), OCTA has been broadly applied in the early detection of vascular abnormalities and diagnosis of vascular pathology of many inherited retinal dystrophies, including RP.^[13]^ It can also be used to provide a quantitative assessment of the microcirculation and microvasculature of the retina and choroid in various layers.^[14]^ Since RP development and progression are both associated with retinal and choroidal vascular changes (as either a primary or a secondary effect), this technology has the potential to bring forward new information about the pathophysiology of RP, as well as to help clinicians with RP diagnosis and management.

## 4. Applications of OCTA in RP

### 4.1. Terminology in OCTA

Various OCTA algorithms have been used by different commercially available OCTA devices: the split-spectrum amplitude-decorrelation angiography (SSADA) algorithm, the optical microangiography complex (OMAGC) algorithm, the OCT-based microangiography (OMAG) algorithm, the OCTA ratio analysis (OCTARA) algorithm, and the amplitude decorrelation algorithm (Table 1).

**Table 1:** Algorithms used by various OCTA machines

Commercialized OCTA Machines					
	AngioVue	AngioPlex	SSOCT Angio	Spectralis OCTA	AngioScan
Company	OptoVue	Zeiss	Topcon	Heidelberg	Nidex
Algorithm	SSADA	OMAGC	OCTARA	Amplitude decorrelation	Modified OMAG
A-scans/s	70,000	68,000	100,000	85,000	53,000
Image dimensions	3 × 3 mm, 4.5 × 4.5 mm, 6 × 6 mm, 8 × 8 mm,	3 × 3 mm, 6 × 6 mm	3 × 3 mm, 4.5 × 4.5 mm, 6 × 6 mm	10°×10,	3 × 3 mm, 6 × 6 mm, 9 × 9 mm, 12 × 9 mm
	6 × 10 mm			15°×15°,	
				20°×20°,	
				15°×30°	

**Figure 1.**
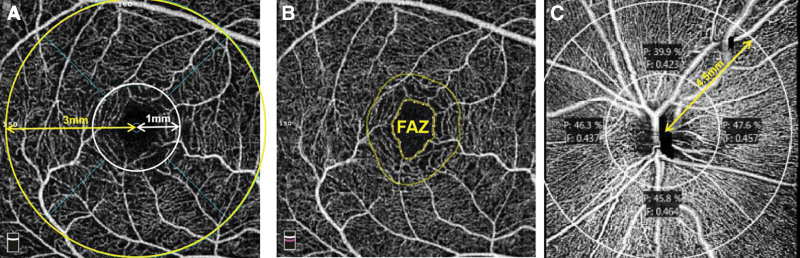
OCTA images of the macular area and the optic disc. (A) OCTA image (OptoVue) of the superficial vascular network centered on the fovea in a healthy subject. (B) OCTA image of the same subject depicting the FAZ area. (C) OCTA image (Zeiss) of the peripapillary area centered on the optic nerve head in a healthy subject. OCTA = optical coherence tomography angiography, FAZ = foal avascular zone.

Several areas of the retina were assessed in the selected studies (Fig. 1). Macular scans were centered on the fovea (Fig. 1A). The “whole image” macular was defined as the whole surface of the scan (generally 3 × 3 or 6 × 6 mm). The fovea was defined as the central 1-mm circle on the macular scan. The parafovea was defined as the central 3-mm circle on the macular scan, except for the fovea, whereas the perifovea was defined as the central 6-mm circle on the macular scan, except for the fovea. The foveal avascular zone (FAZ) was defined as a round capillary-free zone within the macula on OCTA images of the superficial vascular network (Fig. 1B). Optic disc scans were centered on the optic disc (Fig. 1C). The neural canal opening, which terminates the RPE/Bruch membrane complex, was used to define the optic disc area. The peripapillary area was used to describe both the circumpapillary and whole-image peripapillary areas. The whole image peripapillary scan was defined as the entire area of the optic disc scan. Scans assessing the optic disc, the peripapillary area, and the whole image peripapillary area were generally 4.5 × 4.5 mm wide.

### 4.2. OCTA analyses in RP

Thirty-eight published papers evaluating the role of OCTA in RP were identified through a literature search using PubMed. Eighteen cross-sectional clinical studies were chosen after scrutiny (Dr. Lu and Dr. Chiu). For an overview of all these articles, see Table 2.

**Table 2:** Clinical cross-sectional studies using OCTA in RP patients

	Groups and # of Eyes		OCTA Instrument	Scan Area	OCTA Parameter	Summary of the Results			
Article	RP	Control				RP	Control	*P* value	Functional Test
Vessel Density Analysis									
Toto et al. (2016)^[22]^	28 eyes	/	RTVue-XR Avanti	3 × 3 mm (fovea)	Vessel density (SCP)-parafovea	42.2 ± 3.4%	51.4 ± 2.3%	<.001	mfERG
					Vessel density (DCP)-parafovea	42.7 ± 6.2%	56.6 ± 2.2%	<.001	
					Vessel density (CCP)-parafovea	65.3 ± 2.7%	67.2 ± 1.4%	=.024	
Battaglia Parodi et al. (2017)^[20]^	32 eyes	30 eyes	Swept-source OCT DRI Topcon Triton	3 × 3 mm (fovea)	Vessel density (SCP)-parafovea	29.5 ± 6.8%	34.1 ± 4.3%	=.009	/
					Vessel density (DCP)-parafovea	28.7 ± 7.5%	35.5 ± 4.3%	=.001	
					Vessel density (CCP)-parafovea	51.0 ± 4.4%	51.3 ± 2.2%	=.716	
					FAZ area (SCP)	0.277 ± 0.133 mm^2^	0.277 ± 0.133 mm^2^	=.350	
					FAZ area (DCP)	0.541 ± 0.211 mm^2^	0.243 ± 0.157 mm^2^	<.001	
Sugahara et al. (2017)^[23]^	110 eyes	32 eyes	RTVue-XR Avanti	3 × 3 mm (fovea)	Vessel density (SCP)-parafovea	47.0 ± 4.9%	/	=.66	ERG
					Vessel density (DCP)-parafovea	52.4 ± 5.5%	/	<.001	
					Vessel density (CCP)-parafovea	51.0 ± 4.4%	/	=.46	
					FAZ area (SCP)	0.342 ± 0.198 mm^2^	/	=.003	
					FAZ area (DCP)	0.429 ± 0.154 mm^2^	/	=.45	
Mastropasqua et al. (2017)^[32]^	19 eyes	16 eyes	RTVue-XR Avanti	4.5 × 4.5 mm(optic)	RPC vessel density-disc	46.5 ± 7.1%	45.4 ± 10.6%	=.754	/
					RPC vessel density-peripapillary	52.5 ± 5.0%	57.2 ± 5.1%	=.011	
Takagi et al. (2018)^[24]^	50 eyes	22 eyes	RTVue-XR Avanti	3 × 3 mm (fovea)	Flow area (SCP)-parafovea	3.99 ± 0.38 mm^2^	4.32 ± 0.27 mm^2^	=.007	Visual field
					Flow area (DCP)-parafovea	4.06 ± 0.71 mm^2^	4.44 ± 0.37 mm^2^	=.004	
					Flow area (CCP)-parafovea	5.43 ± 0.17 mm^2^	5.47 ± 0.13 mm^2^	=.353	
					FAZ area (SCP)	0.30 ± 0.09 mm^2^	0.36 ± 0.07 mm^2^	=.006	
					FAZ area (DCP)	0.41 ± 0.13 mm^2^	0.42 ± 0.09 mm^2^	=.237	
Koyanagi et al. (2018)^[21]^	73 eyes	36 eyes	RTVue-XR Avanti	3 × 3 mm (fovea)	Flow density (SCP)-fovea	27.1 (11.0–45.8)%	29.1 (22.2–40.1)%	=.309	Visual field
					Flow density (DCP)-fovea	24.5 (8.32–45.8)%	24.7 (17.7–34.1)%	=.757	
					Flow density (SCP)-parafovea	43.8 (34.6–54.6)%	54.7 (41.0–61.1)%	<.001	
					Flow density (DCP)-parafovea	50.1 (39.7–61.1)%	61.7 (55.0–65.5)%	<.001	
					FAZ area (SCP)	0.231 (0.08–1.048) mm^2^	0.225 (0.089–0.371) mm^2^	=0.309	
					FAZ area (DCP)	0.240 (0.085–1.102) mm^2^	0.249 (0.109–0.451) mm^2^	=.890	
Guduru et al. (2018)^[29]^	70 eyes	37 eyes	Swept-source OCT DRI Topcon Triton	6 × 6 mm (fovea)	Flow voids number	55.5 ± 20.1	30.7 ± 16.3	<.01	/
					Flow voids area	0.33 ± 0.12 mm^2^	0.18 ± 0.10 mm^2^	<.01	
Wang et al., 2019^[19]^	40 eyes	26 eyes	Cirrus HD-OCT 5000	3 × 3 mm (fovea)	Vessel area density-fovea	20.5 ± 5.4%	27.5 ± 5.5%	<.01	/
					Vessel area density-temporal	35.5 ± 4.2%	45.1 ± 1.8%	<.01	
					Vessel area density-superior	36.9 ± 3.8%	46.6 ± 1.8%	<.01	
					Vessel area density-inferior	36.7 ± 4.2%	45.9 ± 1.9%	<.01	
					Vessel area density-nasal	36.9 ± 3.8%	45.8 ± 1.8%	<.01	
Hagag et al. (2019)^[28]^	44 eyes	34 eyes	RTVue-XR Avanti	6 × 6 mm (fovea)	Vessel density (SVC)-parafovea				/
					Without CME	68.42 ± 11.27%	65.76 ± 7.4%	=.48	
					With CME	71.19 ± 5.42%	65.76 ± 7.4%	=.005	
					Vessel density (SVC)-perifovea				
					Without CME	65.86 ± 4.7%	65.74 ± 5.24%	=.56	
					With CME	66.07 ± 5.7%	65.74 ± 5.24%	=.83	
					Vessel density (ICP)-parafovea				
					Without CME	47.65 ± 9.71%	50.46 ± 5.65%	=.24	
					With CME	47.89 ± 6.25	50.46 ± 5.65%	=.11	
					Vessel density (ICP)-perifovea				
					Without CME	43.74 ± 7.28	46.73 ± 7.1%	=.14	
					With CME	39.87 ± 4.73	46.73 ± 7.1%	<.001	
					Vessel density (DCP)-parafovea				
					Without CME	21.12 ± 6.84	21.16 ± 4.04%	=.76	
					With CME	20.79 ± 6.76	21.16 ± 4.04%	=.81	
					Vessel density (DCP)-perifovea				
					Without CME	17.74 ± 7.72	25.87 ± 5.71%	<.001	
					With CME	13.10 ± 4.90	25.87 ± 5.71%	<.001	
					Outer retinal thickness-parafovea				
					Without CME	119.15 ± 32.18 µm	147.91 ± 10.53 µm	<.001	
					With CME	79.56 ± 26.66 µm	147.91 ± 10.53 µm	<.001	
					Outer retinal thickness-perifovea				
					Without CME	84.73 ± 28.21 µm	133.08 ± 8.05 µm	<.001	
					With CME	43.79 ± 13.95 µm	133.08 ± 8.05 µm	<.001	
					Inner retinal thickness-parafovea				
					Without CME	192.24 ± 22.10 µm	179.36 ± 10.86 µm	<.08	
					With CME	227.87 ± 33.39 µm	179.36 ± 10.86 µm	<.001	
					Inner retinal thickness-perifovea				
					Without CME	175.58 ± 22.59 µm	150.59 ± 10.44 µm	<.001	
					With CME	188.3 ± 27.47 µm	150.59 ± 10.44 µm	<.001	
Miyata et al. (2019)^[31]^	43 eyes	12 eyes	PLEX Eite 9000	12 × 12 mm (fovea)	Residual choroicapillaris area				Visual field
					Concentric group	44.7 ± 20.2 mm^2^	144.0 ± 0 mm^2^	<.001	
					Vermicular group	124.1 ± 19.1 mm^2^	144.0 ± 0 mm^2^	=.002	
Falfoul et al. (2020)^[25]^	70 eyes	34 eyes	Swept-source OCT DRI Topcon Triton	3 × 3 mm (fovea)	Vessel density (SCP)-parafovea	25.99 ± 5.3%	29.74 ± 3%	=0.0026	mfERG
					Vessel density (DCP)-parafovea	25.04 ± 5.53%	34.47 ± 2.37%	<.001	
					FAZ area (SCP)	369.69 ± 142 µm^2^	312 ± 119 µm^2^	=.1	
					FAZ area (DCP)	575.96 ± 162.94 µm^2^	362 ± 107 µm^2^	<.001	
Corazza et al. (2020)^[30]^	40 eyes	24 eyes	Swept-source OCT DRI Topcon Triton	4.5 × 4.5 mm (fovea)	Vascular density (superficial)	37.23 ± 3.99%	39.28 ± 1.54%	=.0064	MP1
					Vascular density (deep)	38.56 ± 6.3%	42.48 ± 3.66%	=.001	
					Vascular density (choriocapillaris)	49.58 ± 3.43%	51.16 ± 1.38%	=.198	
					Segmentation line 1	44.36 ± 6.78%	51.60 ± 2.91%	=.0003	
					Segmentation line 2	49.70 ± 7.96%	45.14 ± 3.30%	=.15	
					Segmentation line 3	51.13 ± 5.62%	47.54 ± 4.82%	=.848	
Shen et al. (2020)^[1]^	63 eyes	96 eyes	RTVue-XR Avanti;	3 × 3 mm (fovea)	Vessel density (SCP)-parafovea	39.63 ± 6.01%	50.68 ± 3.83%	<.001	Visual field
					Vessel density (SCP)-perifovea	39.63 ± 6.01%	46.65 ± 4.21%	<.001	
				6 × 6 mm (fovea);	Vessel density (DCP)-parafovea	48.10 ± 5.17%	55.03 ± 3.32%	<.001	
					Vessel density (DCP)-perifovea	45.57 ± 5.52%	51.23 ± 5.11%	<.001	
			VG200 SVision Imaging	12 × 12 mm (fovea)	CVI-parafovea	0.25 ± 0.04	0.28 ± 0.06	=.113	
					CVI-perifovea	0.18 ± 0.07	0.25 ± 0.05	=.003	
Macular Microvasculature Analysis									
Inooka et al. (2018)^[35]^	53 eyes	46 eyes	Cirrus HD-OCT 5000	3 × 3 mm (fovea)	Perfusion density (whole)	0.3257 ± 0.0462	0.3895 ± 0.0204	<.001	Visual field
					Perfusion density (superficial)	0.3854 ± 0.0166	0.4166 ± 0.0080	<.001	
					Perfusion density (deep)	0.2929 ± 0.0476	0.3475 ± 0.0298	<.001	
					Vessel length density (whole)	17.566 ± 2.938 mm^−1^	22.034 ± 1.371 mm^−1^	<.001	
					Vessel length density (superficial)	20.205 ± 1.170 mm^−1^	22.646 ± 0.755 mm^−1^	<.001	
					Vessel length density (deep)	14.766 ± 2.711 mm^−1^	18.448 ± 1.769 mm^−1^	<.001	
					Vessel diameter index (whole)	0.0186 ± 0.0006 mm	0.0176 ± 0.0004 mm	<.001	
					Vessel diameter index (superficial)	0.0190 ± 0.0004 mm	0.0184 ± 0.0003 mm	<.001	
					Vessel diameter index (deep)	0.0199 ± 0.0005 mm	0.0188 ± 0.0004 mm	<.001	
					FAZ area	0.3091 ± 0.091 mm^2^	0.2310 ± 0.065 mm^2^	<.001	
Arrigo et al. (2019)^[36]^	32 eyes	32 eyes	Swept-source OCT DRI Topcon Triton	3 × 3 mm (fovea) 4.5 × 4.5 mm (optic)	Vessel density (SCP)-parafovea	0.39 ± 0.02	0.41 ± 0.01	<.01	Visual field
					Vessel density (DCP)-parafovea	0.36 ± 0.03	0.43 ± 0.01	<.01	
					Vessel dispersion (SCP)-parafovea	24 ± 15	11 ± 4	<.01	
					Vessel dispersion (DCP) -parafovea	16 ± 12	11 ± 3	<.01	
					Vessel tortuosity (SCP)-parafovea	4.80 ± 0.29	7.2 ± 0.31	<.01	
					Vessel tortuosity (DCP)-parafovea	4.42 ± 0.49	7.84 ± 0.34	<.01	
					Vessel rarefaction (SCP)-parafovea	0.66 ± 0.04	1.80 ± 0.32	<.01	
					Vessel rarefaction (DCP)-parafovea	0.62 ± 0.03	1.09 ± 0.2	<.01	
Lin et al. (2019)^[33]^	37 eyes	54 eyes	RTVue-XR Avanti	3 × 3 mm (fovea)	Vessel density (SCP)-parafoveal				
					Moderate	41.83 ± 4.56%	48.95 ± 3.73%	<.001	
					Severe	41.64 ± 4.93%	48.95 ± 3.73%	<.001	
					Vessel density (DCP)-parafoveal				
					Moderate	47.7 ± 9.44%	52.84 ± 3.4%	=.026	
					Severe	39.58 ± 9.46%	52.84 ± 3.4%	<.001	
					Cone density-C1				
					Moderate	22726 ± 2648/mm^2^	23691 ± 2941/mm^2^	=.96	
					Severe	16338 ± 4139/mm^2^	23691 ± 2941/mm^2^	<.001	
					Cone density-C2				
					Moderate	19885 ± 2427/mm^2^	23278 ± 2776/mm^2^	<.001	
					Severe	14751 ± 3089/mm^2^	23278 ± 2776/mm^2^	<.001	
					Cone density-C3				
					Moderate	18741 ± 2317/mm^2^	20974 ± 2074/mm^2^	=.005	
					Severe	14091 ± 2863/mm^2^		<.001	
					Cone density-C4				
					Moderate	18086 ± 2086/mm^2^	18734 ± 1460/mm^2^	=.86	
					Severe	13794 ± 3030/mm^2^	18734 ± 1460/mm^2^	<.001	
					Cone density-C5				
					Moderate	17473 ± 2084/mm^2^	17247 ± 1327/mm^2^	=1.00	
					Severe	13428 ± 3034/mm^2^	17247 ± 1327/mm^2^	<.001	
AttaAllah et al. (2020)^[34]^	30 eyes	24 eyes	RTVue-XR Avanti	6 × 6 mm (fovea)	Vessel density (SCP)-parafovea	45.2 ± 3%	46.8 ± 5.2%	=.191	/
					Vessel density (DCP)-parafovea	44 ± 5.8%	55 ± 5.2%	<.001	
					Vessel density (CCP)-parafovea	63.8 ± 2.4%	65.8 ± 1.8%	<.001	
					FAZ area (SCP)	0.5 ± 0.2 mm^2^	0.3 ± 0.1 mm^2^	<.001	
					FAZ area (DCP)	0.6 ± 0.2 mm^2^	0.3 ± 0.1 mm^2^	<.001	
Arrigo et al. (2020)^[37]^	45 eyes	45 eyes	Swept-source OCT DRI Topcon Triton	3 × 3 mm (fovea) 4.5 × 4.5 mm(optic)	Choroidal thickness				/
					Pattern 1 (early stage)	227 ± 37 µm	305 ± 59 µm	=.685	
					Pattern 2 (advanced stage)	218 ± 44 µm	305 ± 59 µm	<.01	
					Pattern 3 (late stage)	156 ± 40 µm	305 ± 59 µm	<.01	
					Choroidal stromal index				
					Pattern 1 (early stage)	0.03 ± 0.01	0.03 ± 0.01	<.01	
					Pattern 2 (advanced stage)	0.07 ± 0.01	0.03 ± 0.01	<.01	
								<.01	

CME = cystoid macular edema; CVI = choroidal vessel index; DCP = deep capillary plexus; FAZ = foveal avascular zone; ICP = intermediate capillary plexus; OCTA = optical coherence tomography angiography; RP = retinitis pigmentosa; SCP = superficial capillary plexus; SVC = superficial vessel capillaries.

### 4.3. Vessel density (VD) analysis in RP

#### 4.3.1. Retinal blood flow.

The pathogenesis of RP is quite complex and is mainly related to genetic alterations at the photoreceptor and RPE cell levels, ultimately leading to retinal degeneration.^[6]^ However, the importance of the involvement of both the inner retina and vascular supply has been increasingly recognized in recent years.^[15]^ Photoreceptor cell loss with the reduction of oxygen consumption has been suggested, and thus decreased the need for oxygen delivery from the retinal circulation in the pathology of eyes with RP. Such changes in oxygen diffusion are assumed to cause attenuation of the vessels.^[16]^ Histopathological studies showed that the features of RP included vessel narrowing and sclerosis, followed by thickening of the blood vessel wall and lumen occlusion.^[17]^ In agreement with these histopathological findings, abundant evidence has proved that vascular changes (e.g. perivascular cuffing, arteriolar attenuation, and reduced ocular blood flow) feature RP which was hypothesized to be part of the pathogenic process with advanced technologies.^[4]^ Reduced retinal blood flow velocity and vascular diameter were demonstrated with the use of magnetic resonance imaging (MRI)^[18]^ and color Doppler flow imaging (CDFI)^[19]^ in RP patients. However, each of these techniques has its limitations, such as being qualitative or invasive.

OCTA parameters have been demonstrated to enable earlier detection of circulatory alterations compared to other conventional methods.^[19]^ Moreover, OCTA has potential advantages over conventional techniques for assessing retinal terminal vessels. Previous studies showed that both SCP and DCP vessel densities are significantly decreased in early-^[20,21]^ and middle- and late-stage RP^[22]^ after comparison with healthy objects. Moreover, a more profound involvement of the deep layer with reduced retinal vasculature signal was found (parafoveal VD: DCP, *P*=.001; SCP, *P*=.009).^[20]^ This finding was later proved by one study that enrolled 110 eyes of RP and 32 control eyes (Parafoveal VD: DCP, *P*<.001; SCP, *P*=.66).^[23]^ Another study demonstrated that the most severe vascular impairment occurred in the parafoveal flow area (DCP, *P*=.004; SCP, *P*=.007).^[24]^ Most recently, one study also showed that vascular alteration in RP might begin at the level of DCP, while the change in the SCP would occur later in the evolution of the disease.^[25]^

However, these conventional OCTA images suffer from projection artifacts, which limit the ability to accurately separate and quantify microvasculature into the three distinct macular vascular layers corresponding to histologic studies^[26,27]^: superficial vascular complex (SVC), intermediate capillary plexus (ICP), and DCP. Advanced projection-resolved OCTA (PR-OCTA) with improved vascular segmentation metrics is required for better visualization and illustration. One study group found that deeper retinal plexuses (both ICP and DCP) were primarily damaged by RP, compared to SVC using the self-developed PR-OCTA algorithm.^[28]^

#### 4.3.2. Choroidal blood flow.

Choroidal vascular features are important in RPE atrophic disorders because of their role in oxygen and nutrition supply as well as metabolic exchanges to the outer retina and RPE. Early histopathology studies have shown the absence of choriocapillaris in RP, which has lost photoreceptors and forms bone-spicule pigments.^[17]^ However, when analyzing the blood flow at the CCP layer, there are controversies in the findings of different studies. Vessel densities of CCP in middle- and late-stage RP patients were reported to be remarkably lower than those in healthy subjects (*P*=.024),^[22]^ while other studies reported no differences in CCP vessel densities between RP patients and normal controls.^[20,24]^ These discrepancies could be explained by the limitations of conventional OCTA devices that suffer from projection artifacts and penetration depth. With advanced OCTA technology, accurate choroidal changes can be detected by SS-OCTA which has a light source of 1050 nm. Guduru et al demonstrated that flow voids (FVs) in patients with RP were significantly reduced compared to healthy subjects (FV area: *P*<.01), indicating that the compromised choriocapillaris might be a result or cause of RP.^[29]^ Manual segmentation lines for OCTA images were added to separate deep choroids at different levels (L1, L2, and L3) from Bruch’s membrane to obtain more precise data on the whole choroid in one study,^[30]^ and a more significant reduction in vascular density was found in L1, which could be explained by subsequent retinal degeneration. Recently, wide-angle OCTA (8 × 8 mm or 12 × 12 mm) has been applied to investigate choriocapillaris defects, for the fact that small angle OCTA (3 × 3 mm or 6 × 6 mm) failed to detect differences in choriocapillaris in RP patients since the peripheral retina is more likely to be affected at the early stage.^[1,31]^

#### 4.3.3. *Blood flow in optic disc and peripapillary area*.

The radial peripapillary capillary (RPC) network is a unique vascular plexus around the optic disc within the retinal nerve fiber layer (RNFL). It originates from the central retinal artery and branches out and runs parallel to the axon direction of the retinal ganglion cells (RGCs). Therefore, it is believed that the blood flow of the RPC, as well as the thickness of the RNFL around the optic disc, is reduced in RP patients, although the specific mechanism has not been clarified. In particular, the biomicroscopic aspect of the “pale” optic disc, together with its typical vascular attenuation, suggests the presence of vascular damage in the optic disc and peripapillary area. With the newly developed OCTA technique, the RPC network can be visualized separately. In one cross-sectional study that included 11 RP patients (22 eyes) and 16 age-matched control subjects (16 eyes), RPC vessel densities in the optic disc and peripapillary areas were found to be significantly reduced in RP patients along with a reduction in RNFL thickness, indicating that vascular changes in these areas might be associated with RGC death and RNFL thinning, which are the two pathological processes accompanying inner retinal disorganization.^[32]^

### 4.4. *Macular microvasculature analysis in RP*

Many studies have suggested that macular microvasculature changes caused by decreased blood flow might be indicated in the development of RP and the pathogenesis of RP in the macular area, particularly in the final loss of central vision. It has been confirmed that there is a reduction in macular vascular density in the eyes of patients with RP, as shown by different types of OCTA devices. Lin *et al*. evaluated the macular structural changes in the parafoveal regions in normal subjects and mild- or late-stage RP patients with objectively quantified cone density (CD) and microvascular density and showed significant cone loss in RP patients.^[33]^ AttaAllah et al demonstrated a reduction in macular microvascular density in all studied layers on OCTA as well as macular structural changes such as EZ disruption and FAZ enlargement.^[34]^ However, findings regarding the FAZ area have been controversial. Parodi et al found that the FAZ area was significantly enlarged only at the level of the DCP in RP eyes.^[20]^ Conversely, Koyanagi et al found a significant enlargement of the superficial, but not deep, FAZ area, which was relatively preserved until the mild-to-late stages.^[21]^

Quantitative OCTA parameters help to identify retinal vascular abnormalities in patients with RP. Measurements of geometric vascular features using OCTA biomarkers may become a useful tool to monitor disease activity and the efficacy of new therapeutic modalities. Inooka et al showed that both qualitative and quantitative changes in microvascular density and morphology are useful for assessing the pathophysiology of RP.^[35]^ With an automated program, indices of the microvascular density, perfusion density (PD), and vessel length density (VLD) were found to be significantly reduced, and the vessel diameter index (VDI) was significantly increased in RP patients. Arrigo et al analyzed the changes in vascular features at the level of both the macula and optic nerve between RP patients and healthy controls.^[36]^ Their results revealed a statistically significant difference in all the calculated OCTA parameters, including vessel density, vessel tortuosity, vessel dispersion, and vessel rarefaction, with VT and VR being the most reliable biomarkers to describe the abnormalities of geometric vascular features in RP patients. In addition, quantitative measurements of choroidal features, including choroidal stromal index (CSI), VT, VDisp, and vessel density, revealed further detailed information regarding the changes in choroidal patterns in RP patients which were found to be associated with different RP clinical forms as well as with different progression after 1 year using SS-OCTA.^[37]^

## 5. Correlation of OCTA parameters with retinal function analysis in RP

Correlation studies between the quantitative vascular OCTA parameters and retinal function (measured either with objective methods such as multifocal ERG or subjective methods such as visual acuity, visual field, and microperimetry) could better clarify whether any functional impairment corresponds to vascular signal changes. Functional dysregulation of retinal and choroidal changes seems to occur in patients with late-stage RP. Toto et al demonstrated that both SCP and DCP vessel densities in the macular region are correlated with the macular function, as well as with the GCC thickness.^[22]^ Liu et al analyzed the correlation of choroidal small/middle and large vessel density with retinal photoreceptor cells and visual function in patients with RP, which demonstrated that choroidal microcirculation was a prominent factor affecting the visual acuity, visual field, and ERG b-wave amplitude in patients with RP.^[38]^ This may provide new insights into the mechanisms and treatment of RP. With the advantage of SS-OCTA, various types of choriocapillaris defects can be defined according to the choroidal vascular structure, and the degree of choriocapillaris defects was correlated with the BCVA, Humphrey indexes, and microperimetry index.^[1]^ Falfoul et al also found a statistically significant correlation between macular function and parafoveal DCP density.^[25]^

## 6. Limitations and future visions for OCTA in RP management

The relationship between retinal and choroidal vascular changes and retinal function should be further confirmed in a larger population. Moreover, the nature of the cross-sectional design of these previous studies makes it insufficient to precisely assess the cause-effect relationship between retinal and choroidal vasculature changes and retinal function. Further prospective studies with longer follow-up periods combining retinal function studies and microvasculature changes will provide a better understanding of the pathophysiology of RP patients.

OCTA is a useful tool for monitoring RP disease progression and may be used to measure retinal vascular parameters as outcomes in clinical trials. It is important, however, to recognize its limitations, such as susceptibility to motion artifacts, projection artifacts, limited comparability among different OCTA devices, and restricted contribution of information regarding the grade of disease activity. With the continuing improvement of OCTA technology, PR-OCTA can enable the clean visualization of some retinal plexuses and vascular pathologies using post-processing algorithms to reduce projection artifacts.^[39]^ The ability of the newly developed wide-field OCTA (WF-OCTA) was also compared to that of ultra-wide-field fluorescein angiography (UWF-FA) and ultra-wide-field color fundus photography (UWF-CP) for retinal disorder detection.^[40]^ Retinal biomarkers from OCTA images can facilitate the clinical management of retinal disorders. However, current commercially available OCTA devices are not able to provide a wide field of quantifiable retinal biomarkers because of the limitations of their analysis software. An “all-in-one” metric that can generate comprehensive retinal biomarkers, including parameters depicting geometric vasculature features with accurate definition, as well as those quantifying blood flows with improved classification and segmentation, for retinal disorder analysis is of great importance and convenience for both clinicians and patients. Hence, we have recently invented a method for establishing computational retinal microvascular biomarkers (CRMBs) through a knowledge-driven computerized automatic analytical system based on fractal analysis using OCTA images (manuscript in preparation for submission). We anticipate that these CRMBs will directly lead to a new classification of RP patients, facilitating better understanding, early detection, timely treatment, and improved quality of life.

## 7. Conclusions

The application of OCTA is beneficial for studying alterations in the retinal vasculature in the progression of RP. In this review, we summarised the current OCTA findings in clinical research on RP and envisioned future advancements in OCTA. We believe that the development of OCTA is a major contribution to advancing ophthalmic imaging. This will help us to better understand the etiology and pathology of RP. It will also facilitate the diagnosis, monitoring, and treatment of RP.

## Acknowledgments

The authors thank Kin Chiu, MD, PhD, from the University of Hong Kong, for reviewing this paper and her helpful comments.

## Author Contributions

Bingwen Lu had full access to all the data in the study and takes responsibility for the integrity of the data and the accuracy of the data interpretation.

Concept and design: Bingwen Lu.

Acquisition, analysis, or interpretation of data: Bingwen Lu.

Drafting of the manuscript: Bingwen Lu.

Critical revision of the manuscript for important intellectual content: All authors.

Supervision: Like Xie
